# Prospective association between dietary magnesium intake and physical performance in older women and men

**DOI:** 10.1007/s00394-022-02808-z

**Published:** 2022-02-04

**Authors:** Lucía Arias-Fernández, Ellen A. Struijk, Francisco Félix Caballero, Rosario Ortolá, Esther García-Esquinas, Fernando Rodríguez-Artalejo, Esther Lopez-Garcia, Alberto Lana

**Affiliations:** 1 Department of Medicine. School of Medicine and Health Sciences, School of Medicine and Health Sciences, Universidad de Oviedo/ISPA, Calle Julián Clavería, s/n, 33006 Oviedo, Spain; 2 Department of Preventive Medicine and Public Health, School of Medicine, Universidad Autónoma de Madrid-IdiPaz and CIBERESP (CIBER of Epidemiology and Public Health), Madrid, Spain; 3 IMDEA-Food Institute, CEI UAM+CSIC, Madrid, Spain

**Keywords:** Diet, Magnesium, Physical functional performance, Older adults

## Abstract

**Purpose:**

Magnesium is a profuse intracellular cation with a key role in muscle function and cellular senescence. The aim was to examine the prospective association between 5 year changes in dietary intake of magnesium and changes in physical performance among older men and women.

**Methods:**

Prospective study conducted over 863 community-dwellers aged ≥ 65 years from the Seniors-ENRICA cohort (Spain). In 2012 and 2017, a validated computerized face-to-face diet history was used to record the consumption of up to 880 foods. From these data, we estimated changes in dietary magnesium intake. The Short Physical Performance Battery (SPPB) was also conducted in both time points and we obtained changes in the score during follow-up, with positive values indicating physical performance improvement.

**Results:**

Over 5 years of follow-up, an increase in magnesium intake was associated with an increment in the SPPB score among older women [multivariate *β* (95% confidence interval): 1.01 (0.49; 1.52), *p*-trend: 0.001]. In addition, changes from non-adherence to adherence to both estimated average requirement and recommended dietary allowance during follow-up period were associated with an increment in SPPB score among older women [1.14 (0.36; 1.92) and 0.84 (0.22; 1.47), respectively]. No significant associations between changes in magnesium intake and changes in SPPB score were observed in men.

**Conclusions:**

Both increase of magnesium intake and change from non-adherence to adherence to dietary reference magnesium intake was prospectively associated with better physical performance among older women, but not among men.

**Supplementary Information:**

The online version contains supplementary material available at 10.1007/s00394-022-02808-z.

## Introduction

Magnesium (Mg) is a very abundant intracellular cation which sustains several basic cellular processes. Among older adults, Mg deficiency is frequent because of the age-related decline in dietary intake combined with alterations in the Mg absorption/excretion balance [1]. There is evidence linking the Mg deficiency to cellular senescence and to an accelerated aging phenotype [2]. Specifically, given that Mg exerts a key role in muscle function, an inadequate dietary intake could have a negative impact on physical performance in the old age [3, 4].

Slowing the progressive decline in physical performance is one of the keys strategies to achieve healthy aging. To the date, in addition to the fact that studies addressing the association between Mg and physical performance among older adults are scarce and conflicting, it is unclear which method is the most ideal to assess the association. A cross-sectional analysis using baseline data from the InCHIANTI study (aging in the Chianti area, Italy) showed, for the first time, that serum Mg concentration was an independent determinant of muscle performance in older adults [5]. But, given there is controversy about whether serum Mg is an appropriate proxy of Mg stores or not, other studies used dietary Mg intake to address the association, which can also lead to more practical conclusions for clinical counselling and public health interventions [6]. Using a dietetic approach, Veronese et al. found that a daily Mg supplementation improved the physical performance of healthy older women who participated in a parallel randomized controlled trial [7]. Nevertheless, a recent study found no effect of Mg intake on changes in physical performance [8]. Moreover, none of these studies accounted for sex differences, although women could be more susceptible to Mg deficiencies than men, because of their higher frequency of musculoskeletal disorders, including osteoporosis, which is known to limit Mg exchange between bone and blood [7, 9].

Therefore, our study aimed to assess the prospective association between 5 year changes in dietary intake of Mg and changes in physical performance among older men and women.

## Methods

### Study design and participants

This was a prospective study using data from the Seniors-ENRICA cohort (Spain), whose methods have been reported elsewhere [10, 11]. This cohort included community-dwelling adults aged 60 years and older, who were recruited in 2008–10. At baseline, information about lifestyle, health status, morbidity and use of health services was collected with a standardized phone interview. Then, two home visits were conducted to perform a physical examination and to obtain data on habitual diet. In 2012 and 2017, two subsequent waves of data collection were performed, using the same procedures, to update baseline information. Given that physical performance, as measured by the Short Physical Performance Battery (SPPB) [12], was first assessed in 2012, analyses for this work were conducted with the 2519 participants in 2012 who were followed-up to 2017.

Written informed consent was given by all study participants. The Seniors-ENRICA cohort was approved by the Clinical Research Ethics Committee of the *La Paz* University Hospital in Madrid (registration number: 2144).

### Study variables

#### Diet history and mineral intake

In 2012 and 2017, a validated computerized face-to-face diet history was used to assess habitual food consumption during the previous year [13]. This instrument included 880 foods and beverages as well as sets of photographs to help participants estimate serving sizes. Daily Mg intakes were estimated using standard Spanish food composition tables [14]. Mineral intake through supplements was not considered due to its low use (< 1%) and the lack of data on the specific type consumed. The validity of the diet assessment was evaluated by comparing information obtained using the diet history with seven 24-h recalls over a period of 1 year among a subsample of participants; the Pearson correlation coefficient was moderately good for Mg intake (*r* = 0.46) [13]. Intake of Mg was adjusted for total energy using the residuals method [15].

#### Physical performance

The SPPB was used, according to protocol of the National Institute on Aging [12], to assess physical function in 2012 and 2017. The SPPB comprises three standardized timed components: standing balance, gait speed and chair stand. To assess the balance, participants were asked to stand in three progressively difficult positions for 10 s each: feet side by side, semi-tandem, and full-tandem position. Then, gait speed was measured as the shortest time (seconds) of two consecutive walks at a normal pace along a corridor of 2.44 m long (8-foot walk), using their assistive device if needed. Finally, the chair-stand test was performed by asking participants to stand up from a chair and sit down five times with no help from their arms. Each of these three tests was scored from 0 (inability to perform the task) to 4 (highest level of performance). Therefore, a global SPPB score was created by adding each component test score, ranging from 0 to 12 (best performance).

#### Other variables

Data on potential confounders of the study association were also collected in 2012 and 2017. We assessed sociodemographic characteristics (sex, age, education level) and health behaviours, including smoking, alcohol consumption, and time spent watching TV as a proxy of sedentariness [11]. Recreational physical activity, measured in metabolic equivalents hours per week (MET-h/w), was assessed with the validated questionnaire used in the EPIC-Spain cohort study [16].

Data on morbidity were also collected. Body Mass Index (BMI) was calculated as measured weight (kg) divided by square height (m), and obesity was defined as BMI ≥ 30 kg/m^2^. Diabetes was defined as fasting plasma glucose ≥ 126 mg/dl or taking antidiabetic therapy [17]. Blood pressure was measured under standardized conditions [18], and hypertension was defined as systolic/diastolic blood pressure ≥ 140/ ≥ 90 mmHg or being under hypertensive drug treatment. Finally, hypercholesterolemia was defined as cholesterol ≥ 200 mg/dl or undergoing lipid-lowering treatment. Participants also reported the following physician-diagnosed diseases: cardiovascular disease (heart failure, heart attack or stroke), musculoskeletal disease (arthritis, osteoarthritis or hip fracture) and cancer.

### Statistical analysis

Of the 2519 participants in the Seniors-ENRICA in 2012, 1185 were lost to follow-up and 196 died up to 2017. Also, we excluded individuals lacking data on Mg intake (*n* = 263), implausible low or high energy intake values (*n* = 2) or without information on any of the rest of study variables (*n* = 10). Therefore, the analyses were conducted with 863 individuals (424 men and 439 women).

We used multivariable linear regression to study the associations between changes in Mg intake and changes in the SPPB score from 2012 to 2017. The dependent variable was the continuous change in SPPB score, resulting from the subtraction of 2017 minus 2012 values. The main independent variables were sex-specific tertiles of Mg intake change between 2012 and 2017: the first tertile reflected an intake decrease, the second tertile a small intake change, and the third tertile an intake increase. First, residual plots were used to check the assumption of linearity and homoscedasticity. Then, two regression models were fitted with the second tertile as reference: (1) the first model was adjusted for baseline SPPB score, age, educational level, baseline physical activity, smoker status, BMI, TV-watching and alcohol intake; (2) the second model was additionally adjusted for baseline morbidity, including diabetes, hypertension, hypercholesterolemia, cardiovascular disease, cancer and musculoskeletal disease. The linear dose–response association (*p* value for trend) was tested by modelling Mg intake as a continuous variable. We also repeated the analyses using a 1 SD-increment of Mg intake change during the 5-year period as the independent variable. To assess the consistency of results, the analyses were stratified by physical activity, musculoskeletal disease, obesity, cardiometabolic disease (diabetes and cardiovascular disease) and alcohol intake; likelihood-ratio tests comparing models with and without interactions terms were used to assess whether results varied across strata.

In addition, we classified participants according to their level of compliance with the current US dietary reference intake for Mg [19], using both the estimated average requirement (EAR) (≥ 350 mg/d for men and ≥ 265 mg/d for women) and the recommended dietary allowance (RDA) (≥ 420 mg/d for men and ≥ 320 mg/d for women). According to these cut-off points, we defined four categories of participants: non-compliers both in 2012 and 2017; those who moved from non-compliance in 2012 to compliance in 2017; those who moved from compliance in 2012 to non-compliance in 2017; and compliers both in 2012 and 2017. Then, we fitted the same regression models described above, using non-compliers in both time points as the reference group. Moreover, to aim for consistency and generalization, we repeated the analyses using the cut-off points from the European Food Safety Authority (EFSA). Given that the EFSA considers that EAR or RDA for Mg cannot be derived for adults, we used its defined adequate intake (AI) (≥ 350 mg/d for men and ≥ 300 mg/d for women) [20].

Sex differences in the association between changes in Mg intake and SPPB scores were tested with likelihood-ratio tests, which compared models with and without cross-product interaction terms. Given that we found a sex interaction (*p* for interaction = 0.02 in the fully-adjusted model) in main analyses, all results are presented for men and women separately.

Analyses were performed with the STATA software, 15.0 version (Stata Corp, College Station, TX). This manuscript follows the Strengthening the Reporting of Observational Studies in Epidemiology (STROBE) recommendations.

## Results

Table 1 shows the main characteristics of participants according to sex and year of data collection. Throughout the follow-up period, there was a decrease in tobacco consumption, daily energy intake and BMI for both sexes. Likewise, the daily intake of Mg was reduced, leading to a lower percentage of compliance for EAR and RDA recommendations in 2017 compared to 2012. Lastly, the prevalence of chronic diseases was higher in 2017 than in 2012 for both men and women. Over the 5-year follow-up period, we identified 453 (52.5%) participants with minimal or no changes in the SPPB score, 202 (23.4%) with a decrease and 208 (24.1%) with an increase in the score. As a result, there was only a slight decrease in the mean SPPB score between 2012 and 2017 in both men and women.

**Table 1:** Characteristics of participants at baseline and at the end of follow-up in lifestyle, magnesium intake, morbidity and SPPB score, by sex

		Women (*n* = 439)			Men (*n* = 424)		
		2012	2017	*p* value^a^	2012	2017	*p* value^a^
Age, year		70.9 (5.36)	75.6 (5.24)	N/A	70.0 (5.38)	74.8 (5.33)	N/A
Primary education or less, *n* (%)		254 (57.9)	–	N/A	162 (38.2)	–	N/A
Physical activity, METs-h/w		18.8 (12.1)	–	N/A	25.8 (17.0)	–	N/A
TV-watching, h/d		19.6 (10.9)	20.4 (11.9)	0.160	17.1 (9.66)	19.3 (11.6)	< 0.001
Current smokers, *n* (%)		17 (3.87)	16 (3.64)	0.01	59 (13.9)	43 (10.1)	< 0.001
Alcohol intake, g/d		4.36 (8.34)	3.66 (8.49)	0.05	15.6 (16.6)	14.1 (15.9)	0.04
Energy intake, kcal/d		1868 (387)	1,692 (290)	< 0.001	2238 (460)	2029 (363)	< 0.001
BMI, kg/m^2^		28.4 (4.89)	27.4 (4.82)	< 0.001	28.5 (3.65)	27.9 (3.63)	< 0.001
Mg intake, mg/d		332 (114)	300 (76.3)	< 0.001	360 (95.8)	339 (77.8)	< 0.001
Mg intake compliance							
	EAR, *n* (%)	310 (70.6)	271 (64.7)	< 0.001	187 (44.1)	156 (36.8)	< 0.001
	RDA, *n* (%)	187 (42.6)	153 (34.9)	< 0.001	81 (19.1)	61 (14.4)	< 0.001
Morbidity							
Diabetes, *n* (%)		56 (12.8)	71 (16.2)	< 0.001	94 (22.2)	109 (25.7)	< 0.001
Hypertension, *n* (%)		245 (55.8)	275 (62.6)	< 0.001	240 (56.6)	273 (64.4)	< 0.001
Hypercholesterolemia, *n* (%)		231 (52.6)	263 (59.9)	< 0.001	200 (47.1)	223 (52.6)	< 0.001
Cardiovascular^b^, *n* (%)		16 (3.64)	32 (7.29)	< 0.001	25 (5.90)	39 (9.20)	< 0.001
Cancer, *n* (%)		7 (1.59)	13 (2.96)	< 0.001	8 (1.89)	27 (6.37)	< 0.001
Musculoskeletal^c^, *n* (%)		256 (58.3)	327 (74.5)	< 0.001	146 (34.4)	207 (48.8)	< 0.001
SPPB, score		8.46 (2.15)	8.32 (2.72)	0.23	9.31 (2.03)	9.27 (2.45)	0.72

Note for continuous variables, mean value (standard deviation) is reported

*SPPB* Short Physical Performance Battery test, *MET* metabolic equivalent, *TV* Television, *EAR* estimated average requirement, *RDA* recommended dietary allowance

^a^Paired *t*-test for continuous variables and McNemar’s test for categorical variables were used for statistical comparison

^b^Cardiovascular diseases include heart failure, heart attack and stroke

^c^Musculoskeletal diseases include arthritis, osteoarthritis and hip fracture

Table 2 shows the associations between changes in daily Mg intake and changes in the SPPB score between 2012 and 2017, overall and by sex. Compared to women with small changes in Mg intake (tertile 2), those who increased their intake over follow-up (tertile 3) showed beneficial changes in the SPPB score. Specifically, a mean increase in Mg intake of 44.2 mg/d from 2012 to 2017 was associated with an increment of 1.01 points (95% confidence interval: 0.49; 1.52) in the SPPB score; the corresponding value for a 1-SD increase in Mg intake was 0.28 (0.01; 0.47) points. However, no association was observed for men. The study associations did not significantly vary across predefined strata (all *p* for interaction > 0.05), though the association tended to be stronger in women with low physical activity, with musculoskeletal disease, without obesity or cardiometabolic disease and abstainers (Supplementary Table 1).

**Table 2:** Changes in the SPPB score (95% confidence interval) between 2012 and 2017 according to sex-specific tertiles^a^ of change in dietary magnesium intake between 2012 and 2017, overall and by sex

	Tertile 1 (intake decrease)	Tertile 2 (intermediate change)	Tertile 3 (intake increase)	*p*-trend	Per 1-SD increase during 5 year period^b^
Overall					
Participants, *n* (%)	289 (33.5)	287 (33.3)	287 (33.2)		
Change in Mg intake, mg/d	− 109.6 (66.7)	− 22.8 (49.1)	54.5 (49.2)		
Multivariable model 1^c^	0.03 (− 0.32;0.39)	Ref	0.40 (0.05;0.76)	0.042	0.13 (− 0.02; 0.27)
Multivariable model 2^d^	0.04 (− 0.31; 0.39)	Ref	0.43 (0.07; 0.78)	0.034	0.13 (− 0.02; 0.28)
Women					
Participants, *n* (%)	147 (33.5)	146 (33.3)	146 (33.3)		
Change in Mg intake, mg/d	− 130.4 (76.6)	− 30.1 (15.4)	44.2 (48.5)		
Multivariable model 1^c^	0.11 (− 0.40; 0.62)	Ref	1.00 (0.48; 1.51)	0.001	0.34 (0.13; 0.56)
Multivariable model 2^d^	0.09 (− 0.41; 0.61)	Ref	1.01 (0.49; 1.52)	0.001	0.35 (0.14; 0.57)
Men					
Participants, *n* (%)	142 (33.5)	141 (33.3)	141 (33.3)		
Change in Mg intake, mg/d	− 88.05 (45.8)	− 15.2 (15.3)	65.2 (47.7)		
Multivariable model 1^c^	0.02 (− 0.17; 0.50)	Ref	− 0.16 (− 0.65; 0.34)	0.494	− 0.11 (− 0.32; 0.09)
Multivariable model 2^d^	0.05 (− 0.44; 0.54)	Ref	− 0.09 (− 0.59; 0.41)	0.592	− 0.10 (− 0.30; 0.11)

*SPPB*Short Physical Performance Battery test

^a^Sex-specific tertile cut-points were − 59.8 and − 5.99 mg/d in women and − 42.6 and 11.6 mg/d in men for magnesium

^b^1-SD increase = 89.2 mg/d in women and 73.9 mg/d in men

^c^Adjusted for baseline SPPB score (tertiles of score), age (< 70, 70–79, ≥ 80 y), educational level (primary, secondary, university) and physical activity (tertiles of METs-h/w), smoking status (current, former, never), BMI (tertiles of kg/m^2^), TV-watching (tertiles of h/wk) and alcohol intake (tertiles of g/d)

^d^Additionally adjusted for baseline morbidity, including diabetes, hypertension, hypercholesterolemia, cardiovascular disease, cancer, and musculoskeletal disease

Table 3 shows the associations between changes in adherence to the Mg reference intake and changes in SPPB score from 2012 to 2017 stratified by sex. In general, women who became compliant with the EAR between 2012 and 2017 had an increased SPPB score, in comparison with those who were no-compliant in both time points. Specifically, an improvement in EAR adherence (from non-compliance to compliance) was associated with a 1.14 (95% CI 0.36; 1.92) point increase in SPPB. Also, an improvement in the adherence to RDA (from non-compliance to compliance) was associated with an increase of 0.84 (0.22; 1.47) points on the score in women. Again, no associations were observed in men. Similar results were observed when we used the AI from EFSA to define cut-off points for Mg intake recommendations (Supplementary Table 2).

**Table 3:** Changes in the SPPB score (95% confidence interval) between 2012 and 2017 according to compliance with the US dietary reference intake for magnesium between 2012 and 2017, overall and by sex

	Consistent non-compliance^a^	From non-compliance to compliance^a^	From compliance to non-compliance^a^	Consistent compliance^a^
Estimated average requirement				
Overall				
Participants, *n* (%)	235 (27.2)	131 (15.2)	201 (23.3)	296 (34.3)
Mean change, g/d	− 21.4 (42.5)	50.1 (65.4)	− 68.7 (67.4)	− 34.8 (99.2)
Multivariable model^b^	Ref	0.46 (− 0.01;0.92)	− 0.42 (− 0.83; − 0.01)	0.08 (− 0.30; 0.46)
Women				
Participants, *n* (%)	67 (15.3)	62 (14.1)	101 (23.0)	209 (47.6)
Mean change, g/d	− 34.1 (38.0)	36.6 (58.7)	− 76.4 (70.0)	− 44.9 (102.4)
Multivariable model^b^	Ref	1.14 (0.36; 1.92)	− 0.12 (− 0.82; 0.58)	0.55 (− 0.07; 1.18)
Men				
Participants, *n* (%)	168 (39.6)	69 (16.3)	100 (23.6)	87 (20.5)
Mean change, g/d	− 16.4 (43.2)	62.2 (69.0)	− 60.9 (64.1)	− 10.5 (86.7)
Multivariable model^c^	Ref	0.06 (− 0.54; 0.67)	− 0.33 (− 0.86; 0.21)	0.04 (− 0.51;0.61)
Recommended dietary allowance				
Overall				
Participants, *n* (%)	494 (57.2)	101 (11.7)	155 (18.0)	113 (13.1)
Mean change, g/d	− 17.3 (51.1)	66.1 (67.4)	− 104.3 (93.5)	− 40.3 (93.7)
Multivariable model^b^	Ref	0.45 (− 0.02; 0.91)	0.07 (− 0.33; 0.47)	− 0.07 (− 0.51; 0.38)
Women				
Participants, *n* (%)	184 (41.9)	68 (15.5)	102 (23.2)	85 (19.4)
Mean change, g/d	− 29.3 (42.2)	52.8 (62.0)	− 112 (100.5)	− 46.0 (94.1)
Multivariable model^b^	Ref	0.84 (0.22; 1.47)	0.19 (− 0.37; 0.74)	0.34 (− 0.25; 0.93)
Men				
Participants, *n* (%)	310 (73.3)	33 (7.80)	53 (12.5)	28 (6.60)
Mean change, g/d	− 10.2 (54.5)	93.7 (71.1)	− 89.6 (77.1)	− 23.0 (92.2)
Multivariable model^b^	Ref	0.02 (− 0.75; 0.79)	0.13 (− 0.49; 0.76)	− 0.28 (− 1.10; 0.55)

*SPPB* Short Physical Performance Battery test

^a^Cut-off points for recommendations compliance were ≥ 350 mg/d (women) and ≥ 265 mg/d (men) for Estimated Average Requirement, and ≥ 420 mg/d (women) and ≥ 320 mg/d (men) for Recommended Dietary Allowance

^b^Adjusted for baseline SPPB score (tertiles of score), age (< 70, 70–79, ≥ 80 y), educational level (primary, secondary, university) and physical activity (tertiles of METs-h/w), smoking status (current, former, never), BMI (tertiles of kg/m^2^), TV-watching (tertiles of h/wk) and alcohol intake (tertiles of g/d)

## Discussion

In this prospective study of community-dwelling older adults followed over a 5-year period, an increase of dietary Mg intake was associated with an improvement of physical performance, assessed by the SPPB, in women but not in men. Consistently, a change from non-compliance to compliance with the reference intake resulted in a better SPPB score among women.

### Magnesium intake and physical performance

Our results are in line with those from the single randomized controlled trial conducted to the date that investigated if Mg supplementation improves physical performance. Veronese et al. [7] recruited 124 healthy women > 65 years attending to a mild fitness program, and then compared with a group than received supplementation with 300 mg/d of bioavailable Mg with a placebo group. After 12 weeks, women from the treatment group experienced a significant increment of 0.41 points in the SPPB score, compared to women under placebo. In our study, women in tertile 3 of change in Mg intake achieved even larger positive changes in the SPPB score over 5 years (0.98 points). Previously, Lukaski et al. [21] had examined the effects of Mg depletion induced by dietary restriction on physiologic responses during submaximal exercise in ten postmenopausal middle-aged women (45–71 years old). They found that women fed with 180 versus 320 mg/d had worse cardiovascular function during submaximal work, supporting the current RDA for Mg. Moreover, findings from previous observational research are in the same direction. Two large studies with the EPIC-Norfolk and the UK-Biobank cohorts, found a clear cross-sectional positive dose–response between Mg intake and muscle mass [22, 23], which is a strong determinant of physical performance. Other studies have also shown that sarcopenic older adults consumed less Mg than non-sarcopenic ones [24, 25].

Nevertheless, serum Mg does not seem to correlate well with Mg intakes [6]. Indeed, supplementation with dietary Mg increases serum Mg only among subjects with low basal circulating Mg [26]. This is consistent with our study findings. On one hand, the positive effects of dietary Mg were particularly observed in women moving from baseline low levels of intake to an appropriate Mg intake at the end of follow-up (i.e. above EAR or RDA). On the other hand, we found that participants reducing Mg intake during follow-up preserved physical performance if they continued to meet with dietary reference intake, suggesting that supplementation with Mg or dietary modifications to increase serum Mg are only necessary when older adults consume Mg bellow EAR or RDA.

The mechanisms of the effect of Mg on muscle mass or function are only partially known. Dietary Mg regulates some key mechanisms of muscle function, including protein and ATP synthesis, glycogenolysis, fatty acid oxidation, oxygen consumption and electrolyte balance [4]. It has also been postulated that oxidative stress and alterations in calcium homeostasis triggered by Mg reductions can lead to muscle cells damage [27–29]. Furthermore, Mg may exert certain protection against inflammation, which has been associated with an acceleration of age-related loss of skeletal muscle [4, 28, 29]. Moreover, Mg stimulates the process of bone development and mineralization through several mechanisms [28], and there is strong evidence of the positive role of Mg on fracture risk reduction and on bone mineral density [29], which is essential for physical performance [30]. Lastly, results from a recent analysis of US National Health and Nutrition Examination Survey (NHANES) suggested that high Mg intake may have a positive role in cognitive function [31]; thereby, physical performance improvement could be parallel to cognitive impairment prevention.

### Sex differences

The reasons why in our study women but not men benefited from an increase in Mg intake are unknown but could be related to biological differences between sexes. First, there is some evidence of the beneficial effect of Mg on several highly prevalent chronic morbid conditions, such as cardiovascular disease or type 2 diabetes [32–34], which are also associated to physical performance [35, 36]. Given that the positive effects of Mg on cardiometabolic health seem to be greater among women [32, 33], our results could partially be due to beneficial effect of Mg preventing the development of cardiovascular diseases and type 2 diabetes. Indeed, in our sample, the prevalence of these diseases in women were lower than in men. Second, postmenopausal women are affected more often than men from musculoskeletal disorders and sarcopenia [37, 38]. These health conditions, specially osteoporosis, lead to an imbalance in bone deposition and resorption, altering the serum Mg level and making subjects more sensitive to dietary supplements [38, 39]. This is consistent with our findings from stratified analyses, as the observed effect of Mg intake on physical performance tended to be stronger among women with musculoskeletal diseases. Third, Mg is potentially important for the metabolism of anabolic–androgenic steroids and other sex hormones during aging, such as testosterone, progesterone and insulin‐like growth factor 1 [40]. Some studies conducted only with men have found that Mg acts on the muscle via the endocrine system [41]. Given that the levels of these hormones are sex-dependent, it can be expected that the effects of Mg intake on muscle differ between sexes. Nevertheless, given that hormonal decline is steep during ageing, the endocrine pathway would be more plausible among younger subjects. More research is clearly needed to understand why dietary Mg intake improves physical performance only in women and, from a broader perspective, to understand the suggested sex-dependent effects of Mg intake on healthy ageing [39, 42].

### Practical implications

In our longitudinal study, an overall reduction in Mg intake was observed, even among those who were consistently compliant with dietary recommendations. It seems clear that the risk of Mg deficiency is hanging over most of the people during aging process. Therefore, it could be advised a periodically assessment of available Mg to early detect older adults moving to levels below the recommended Mg thresholds. However, there are still no valid and simple methods to determine the available Mg. For instance, serum Mg only represents 1% of total body magnesium, which is mainly intracellular [6, 43]. Thereby, clinical assessment of patients at risk for Mg deficiency could be actually recommended, which may include the routine evaluation of Mg intake. Given that it is unrealistic to ask clinicians to perform a complete diet history to estimate Mg intake, they could be requested to inquire and counsel about the consumption of main food sources Mg. These sources include foods who are usually part of most healthy diet patterns, such as whole grain cereals, vegetables, nuts, and legumes [43]. For instance, in our cohort of Spanish older adults, main sources of Mg were cereals, dairy products and nuts. Conversely, ultra-processed foods, which are predominant in the Western diet, are normally poor in Mg [43].

### Strengths and limitations

Among the strengths of our study is a rather large sample size, a long follow-up that allows to detect substantial changes in physical performance, and a separate data analyses in men and women. Also, we used a validated diet history to ascertain changes in Mg dietary intake across the follow-up period. Lastly, statistical analyses were adjusted for a good number of potential confounders and included several sensitivity analyses to provide robustness. Nevertheless, this study also has some limitations. First, as in any nutritional epidemiological study, there could be some recall bias; however, this type of bias usually tends to underestimate study associations. Second, although having information on serum Mg levels would have been desirable, studies have shown a lack of correlation between Mg intake and its serum levels suggesting that the latter should not be used to predict disease risk [44]. Third, although we used musculoskeletal disorders as a proxy of bone health, we lacked data about osteoporosis, which is closely related to minerals and physical performance. Finally, despite the prospective nature of the study reverse causality cannot completely be ruled out, since participants might have first experimented a deterioration in physically performance and later, a decrease in their capacity to buy and cook a healthy diet with the appropriate amount of magnesium. Nevertheless, if this was the actual direction of causality, we would have expected the same results for women and men.

### Conclusions

An increase of dietary Mg intake was prospectively associated with better physical performance in older women. Adhering to reference Mg intake in the old age could help women to maintain physical functioning and delay the disability process. Further studies are needed to confirm and understand why this association might occur exclusively in women.

## Supplementary Information

Below is the link to the electronic supplementary material.

Supplementary file1 (DOCX 21 KB)

## Data Availability

Lucía Arias-Fernández, Ellen A Struijk, Francisco Félix Caballero, Rosario Ortolá, Esther García-Esquinas, Fernando Rodríguez-Artalejo, Esther Lopez-Garcia, and Alberto Lana comply with the Ethical guidelines for authorship and publishing in the European Journal of Nutrition.

## Abbreviations

Mg: Magnesium
SPPB: Short Physical Performance Battery
BMI: Body mass index
EAR: Estimated Average Requirement
RDA: Recommended Dietary Allowance
EFSA: European Food Safety Authority
AI: Adequate intake
NHANES: National Health and Nutrition Examination Survey
