# Supplementary material for: Prospective association between dietary magnesium intake and physical performance in older women and men
Source: Eur J Nutr. 2022 Feb 4;61(5):2365–73. doi: 10.1007/s00394-022-02808-z (PMC9279200; doi:10.1007/s00394-022-02808-z)
Supplement: Supplementary file 1 — Supplementary file1 (DOCX 21 KB) [file 394_2022_2808_MOESM1_ESM.docx]

**Supplementary table 1.** Changes in the SPPB score (95% confidence interval) between 2012 and 2017 according to sex-specific tertiles^a^ of change in dietary magnesium intake between 2012 and 2017, by sex. Adjusted models^b^ stratified by baseline physical activity, musculoskeletal disease, obesity, cardiometabolic disease and alcohol intake (N=863).

|  | | Tertile 1  (Intake decrease) | Tertile 2  (Intermediate intake) | Tertile 3  (Intake increase) | P for interaction |
| --- | --- | --- | --- | --- | --- |
| Women | |  |  |  |  |
|  | Physical activity ≤median^c^ | 0.18 (-0.56; 0.92) | Ref. | 0.97 (0.24; 1.70) | 0.79 |
|  | Physical activity >median^c^ | -0.14 (-0.89;0.61) | Ref. | 1.07 (0.30; 1.83) |  |
|  | With musculoskeletal disease^d^ | 0.71 (0.06; 1.36) | Ref. | 1.43 (0.77; 2.09) | 0.02 |
|  | Without musculoskeletal disease^d^ | -0.76 (-1.61; 0.94) | Ref. | 0.31 (-0.55; 1.16) |  |
|  | BMI≥30 kg/m^2^ | -0.27 (-1.19; 0.64) | Ref. | 0.82 (-0.06; 1.70) | 0.07 |
| . | BMI<30 kg/m^2^ | 0.26 (-0.39; 0.90) | Ref. | 1.09 (0.43; 1.74) |  |
|  | With cardiometabolic disease^e^ | -0.24 (-1.98; 1.50) | Ref. | -0.47 (-2.15; 1.21) | 0.07 |
|  | Without cardiometabolic disease^e^ | 0.06 (-0.49; 0.60) | Ref. | 1.16 (0.60; 1.72) |  |
|  | Moderate/heavy drinker | -0.07 (-0.75; 0.61) | Ref | 0.68 (-0.03; 1.38) | 0.45 |
|  | Abstainer^f^ | 0.30 (-0.45; 1.06) | Ref | 1.40 (0.66; 2.14) |  |
| Men | |  |  |  |  |
|  | Physical activity ≤median^c^ | -0.37 (-1.14;0.39) | Ref. | -0.40 (-1.19;0.40) | 0.40 |
|  | Physical activity >median^c^ | 0.39 (-0.25;1.03) | Ref. | 0.18 (-0.45;0.81) |  |
|  | With musculoskeletal disease^d^ | 0.17 (-0.79; 1.14) | Ref. | -0.19 (-1.14; 0.76) | 0.57 |
|  | Without musculoskeletal disease^d^ | 0.09 (-0.65; 0.39) | Ref. | 0.07 (-0.52; 0.66) |  |
|  | BMI≥30 kg/m^2^ | 0.51 (-0.34; 1.35) | Ref. | 0.26 (-0.61; 1.13) | 0.75 |
|  | BMI<30 kg/m^2^ | -0.05 (-0.68; 0.58) | Ref. | -0.16 (-0.80; 0.48) |  |
|  | With cardiometabolic disease^e^ | 1.25 (0.12; 2.37) | Ref. | 0.69 (-0.40; 2.37) | 0.08 |
|  | Without cardiometabolic disease^e^ | -0.32 (-0.87; 0.23) | Ref. | -0.32 (-0.88; 0.24) |  |
|  | Moderate/heavy drinker | 0.04 (-0.49; 0.57) | Ref. | -0.21 (-0.75; 0.33) | 0.44 |
|  | Abstainer^f^ | 0.55 (-0.98; 2.08) | Ref. | 0.47 (-0.99; 1.94) |  |

Note SPPB: Short Physical Performance Battery test; BMI: body mass index: CVD: cardiovascular disease

^a^Sex-specific tertile cut-points were -59.8 and -5.99 mg/d in women and -42.6 and 11.6 mg/d in men for magnesium.

^b^Model adjusted for baseline SPPB score (tertiles of score), age (<70, 70-79, ≥80 y), educational level (primary, secondary, university) and physical activity (tertiles of METs-h/w), smoking status (current, former, never), BMI (tertiles of kg/m^2^), TV-watching (tertiles of h/wk) and alcohol intake (tertiles of g/d)

^c^Sex-specific median for physical activity were 23.5 METs-h/wk for men and 17.5 METs-h/w for women.

^d^Musculoskeletal disease included arthritis, osteoarthritis or hip fracture.

^e^Cardiometabolic disease included cardiovascular disease (heart failure, heart attack and stroke) and diabetes.

^f^Abstainer category included consumers of <0.1 g/d of alcohol

**Supplementary table 2**. Changes in the SPPB score (95% confidence interval) between 2012 and 2017 according to EU dietary reference values for magnesium (N=863)

|  |  |  | Consistent non-compliance^a^ | From non-compliance to compliance^a^ | From compliance to non-compliance^a^ | Consistent compliance^a^ |
| --- | --- | --- | --- | --- | --- | --- |
| Adequate intake | | |  |  |  |  |
|  | Overall | |  |  |  |  |
|  |  | Participants, n (%) | 313 (36.3) | 135 (15.6) | 203 (23.5) | 212 (24.6) |
|  |  | Mean change, g/d | -21.8 (41.6) | 35.7 (65.7) | -83.4 (86.0) | -28.6 (92.3) |
|  |  | Multivariable model^b^ | Ref. | 0.58 (0.14; 1.02) | -0.05 (-0.44; 0.33) | 0.19 (-0.20; 0.57) |
|  | Women | |  |  |  |  |
|  |  | Participants, n (%) | 145 (33.0) | 66 (15.0) | 103 (23.5) | 125 (28.5) |
|  |  | Mean change, g/d | -28.2 (38.9) | 44.7 (61.3) | -105.2 (98.4) | -41.2 (94.3) |
|  |  | Multivariable model^b^ | Ref. | 1.09 (0.44; 1.75) | 0.32 (-0.25; 0.90) | 0.54 (-0.01; 1.09) |
|  | Men | |  |  |  |  |
|  |  | Participants, n (%) | 168 (39.6) | 69 (16.3) | 100 (23.6) | 87 (20.5) |
|  |  | Mean change, g/d | -16.4 (43.2) | 62.2 (69.0) | -60.9 (64.2) | -10.5 (86.7) |
|  |  | Multivariable model^b^ | Ref. | 0.06 (-0.54; 0.67) | -0.33 (-0.85; 0.21) | 0.05 (-0.51; 0.61) |

Note SPPB: Short Physical Performance Battery test

^a^Cut-off points for recommendation compliance were ≥300 mg/d (women) and ≥350 mg/d (men).

^b^Model adjusted for baseline SPPB score (tertiles of score), age (<70, 70-79, ≥80 y), educational level (primary, secondary, university) and physical activity (tertiles of METs-h/w), smoking status (current; former; never), BMI (tertiles of kg/m^2^), TV-watching (tertiles of h/wk) and alcohol intake (tertiles of g/d)
